# Application of two-parameter scoliometer values for predicting scoliotic Cobb angle

**DOI:** 10.1186/s12938-017-0427-7

**Published:** 2017-12-04

**Authors:** Hsuan-Hsiao Ma, Ching-Lung Tai, Lih-Huei Chen, Chi-Chien Niu, Wen-Jer Chen, Po-Liang Lai

**Affiliations:** 1 Department of Orthopaedics and Traumatology, Veterans General Hospital, Taipei, Taiwan; 2 Graduate Institute of Medical Mechatronics, Department of Mechanical Engineering, Chang Gung University, Taoyuan, Taiwan; 3 Department of Orthopaedic Surgery, Bone and Joint Research Center, Chang Gung Memorial Hospital at Linkou, 5 Fushing St. Kweishan, Taoyuan, 33305 Taiwan; 4 College of Medicine, Chang Gung University, Taoyuan, Taiwan

**Keywords:** Cobb angle, Idiopathic scoliosis, Nash–Moe rotation, Rib hump, Scoliometer

## Abstract

**Background:**

Adolescent idiopathic scoliosis, in which obvious curves are visible in radiographic images, is also seen in combination with lumps in the back. These lumps contribute to inclination, which can be measured by a scoliometer. To the authors’ knowledge, there are no previous formulas combining thoracic and lumbar scoliometer values simultaneously to predict thoracic and lumbar Cobb angles, respectively. This study aimed to create more accurate two-parameter mathematical formulas for predicting thoracic and lumbar Cobb angles.

**Methods:**

Between Dec. 2012 and Jan. 2013, patients diagnosed with idiopathic scoliosis in an outpatient clinic were enrolled. The maximal trunk rotations at the thoracic and lumbar regions were recorded with a scoliometer. Right asymmetry hump was deemed positive (+), and left asymmetry hump was deemed negative (−). The Cobb angles were measured with a Picture Archiving and Communication System. Statistical analysis included Pearson’s correlation coefficient, multivariate regression and Bland–Atman analysis.

**Results:**

One-hundred and one patients were enrolled in our study. The average thoracic curve (TC) was 23.3 ± 1.8°, while the average lumbar curve (LC) was − 23.3 ± 1.4°. The thoracic inclination (TI) and lumbar inclination (LI) were 4.5 ± 0.7 and − 5.9 ± 0.6, respectively. The one-parameter formula for the thoracic curve was TC = 2.0 TI + 14.3 (r = 0.813); for the lumbar curve, it was LC = 0.9 LI − 16.9 (r = 0.409). By multivariate regression, the two-parameter formulas for the thoracic and lumbar curves were TC = 2.6 TI − 1.4 LI (r = 0.931) and LC = − 1.5 TI + 2.0 LI (r = 0.874), respectively. The two-parameter formulas were more accurate than the one-parameter formulas.

**Conclusions:**

Based on the results of these two-parameter formulas for thoracic and lumbar curves, the Cobb angles can be predicted more accurately by the readings of the scoliometer. Physicians and other healthcare practitioners can thus evaluate patients with scoliosis more precisely than before with a scoliometer.

## Background

Scoliosis screening has been practiced worldwide for several decades and has offered dependable data about the prevalence, etiology and natural course of idiopathic scoliosis [1]. Adolescent idiopathic scoliosis (AIS), in which abnormal structural curvature of the spine is the exclusive diagnosis, is reported only when other causes of scoliosis have been ruled out [2]. The gold standard to diagnose scoliosis is through radiographic examination, although several studies have indicated a relationship with surface back deformities measured by topography [3], a scoliometer, an integrated shape imaging system [4], and other methods. In terms of school screening, not only is radiographic examination expensive, but parents also worry about their children being exposed to too much radiation.

Vertebral rotations with subsequent rib deformity will cause trunk asymmetry. Adam’s bending test will display more prominent back hump. The Nash–Moe method [5] is one of the methods used to assess the extent of vertebral body rotations by plain radiography. Pedicles of the vertebrae will shift to one side, and the vertebral alignment will change to a convex curve, which is shown in radiographs. The pedicles in the Nash–Moe method ostensibly offer better visibility of the selected anatomical landmark over a greater range of angles. Moreover, vertebral body rotations cause the rib cage hump due to the joints connecting vertebrae and ribs, which can be detected by a scoliometer.

The scoliometer, a tool to measure the angle of trunk rotation, was first promoted by Bunnel in 1984 [6]. Patients with scoliosis may exhibit rib cage deformity associated with rib humps [7]. Measurement of the rib inclination with a scoliometer is performed to estimate the rotational deformity in the transversal plane of the body [8]. Rib prominence on forward flexion during Adam’s forward bending test can be measured by a scoliometer [9]. However, estimating the approximate degree of the scoliosis Cobb angle, which is measured by radiographic plane film, with the use of a scoliometer is impossible because the scoliometer measures only the axial trunk inclination. Nevertheless, in 1996 Korovessis et al. constructed a mathematical formula demonstrating that a scoliometer combined with the formula not only allows the detection of scoliosis but also significantly aids in follow-up observation [10]. In 2003, Sapkas et al. also reported mathematical formulas to predict Cobb angles by scoliometer [11]. In 2015, Prowse et al. performed a systematic analysis of the accurate and reproducible methods to predict scoliotic curvature. Moderate evidence existed for the use of a scoliometer with a mathematical formula [12], but the data are still limited.

The purpose of this study was to identify the relationship between back inclination measured by a scoliometer and Cobb angle measured by X-ray. In addition, we assumed that the degree of thoracic curvature and that of the lumbar curve can influence one another. One parameter (either thoracic inclination or lumbar inclination) and two parameters (both thoracic inclination and lumbar inclination) can be used to create mathematical formulas to predict the Cobb angle using scoliometer measurements. Moreover, we assumed that two parameters are more accurate because the inclinations of both thoracic and lumbar hump are influenced simultaneously by thoracic and lumbar rotation. X-ray has the disadvantage of radiation exposure. If Cobb angles can be predicted according to scoliometer values, this may help to reduce the X-ray exposure in the subsequent follow-up. To the best of our knowledge, this is the first paper which uses two-parameters readouts to predict Cobb angles.

## Methods

We enrolled outpatient clinic patients from Dec. 2012 to Jan. 2013. The study was approved by the Institutional Review Board (CGMH-IRB104-5783B). The inclusion criteria were age between 8–20 years, no radiographic signs of congenital deformity, no limb discrepancy, no active spine disease (postoperative scoliosis, disc disease, sciatica, infection, neuromuscular scoliosis, etc.), and no trauma history of the spine or chest. Subsequently, we collected clinical parameters including apical thoracic and lumbar scoliometer value, age, and sex. Radiographic parameters included scoliotic Cobb angle and Nash–Moe rotation. All values were expressed as the mean ± standard error.

### Nash–Moe method

The Nash–Moe method divides the extent of apical vertebral rotation into 5 grades. At Grade 0, pedicle shadows are equidistant from the sides of the vertebra. The pedicle shadow on the convexity that has moved from the edge of the vertebral body is defined as Grade 1. Grade 3 is defined as the pedicle shadow being in the middle of the vertebral body, and Grade 2 is between Grade 1 and Grade 3. In Grade 4, the pedicle shadow passes through the middle of the vertebra [13].

### Scoliometer

**Fig. 1:**
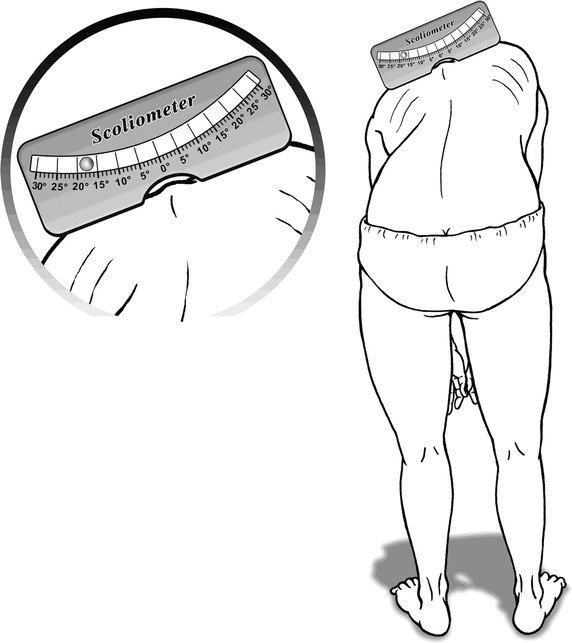
The inclination is measured by placing the scoliometer on the back hump according to Adam’s forward bending test

Before placing the scoliometer on the back, the patients were asked to do a standing forward bend. In this position, they looked down and kept their feet apart, their shoulders loose, and knees extended, and put their hands in front of their knees with their elbows straight and palms opposed [14]. A Scoliometer^®^ (Orthopedic Systems Inc., Mizuho Ikakogyo Co., Ltd., Tokyo, Japan) was then used at two areas of interest: one at the thoracic hump and the other at the lumbar hump (Fig. 1). The senior author obtained scoliometer measurements over the most prominent thoracic and lumbar curves, respectively. The right hump assigned the thoracic and lumbar inclination a positive value, while the left hump assigned the thoracic and lumbar inclination a negative value.

### Cobb angle

Cobb angles were measured by a semi-automated method using a Picture Archiving and Communication System (PACS, Centricity, GE-Healthcare). The most tilted vertebrae above and below the apex of the curve were chosen. Tangential lines were drawn from the superior end plate of the superior vertebra and the inferior end plate. The angle calculated by PACS between intersecting lines was the Cobb angle [15, 16]. Moreover, the degree of the Cobb angle was defined as positive (+) when the curve angled toward to the right and as negative (−) when it angled to the left.

### Data analysis and mathematical formula

The data were analyzed using SPSS v.17 (SPSS Version 17.0, Chicago). Box-plot graphs were presented between the classification of Nash–Moe rotation and each inclination. In addition, 95% confidence intervals were also shown in the graphs. For a single parameter, we assumed the formula y = ax + b, where y represented the Cobb angle and x was the respective inclination. We obtained a and b by simple linear regression. For two parameters, the assumed formula was y = ax_1_ + bx_2_ + c, where y represented the thoracic or lumbar Cobb angle. The variable “c” was set at 0 to ensure that y was 0 if both x_1_ and x_2_ were 0. We obtained a and b by multiple regression analysis. The coefficient value was statistically significant when the *p* value was < 0.05. For all subjects, Bland–Atman plots was used to demonstrate agreement between the Cobb angles estimated by the two-parameter formulas and the Cobb angles measured from the radiographs. The difference of Cobb angle values between the two methods was plotted against the average of the two methods. 95% of the data points lies within ± 2.0 SD was considered agreement between two methods.

## Results

### Demographic data

There were 101 patients (82 females, 19 males) enrolled in this study. The mean age was 13.9 ± 0.2 years and the mean body mass index was 18.6 ± 0.3. The average radiographically measured Cobb angle of thoracic curvature was 23.3 ± 1.8°, while the average thoracic inclination measured by scoliometer was 4.5 ± 0.7°. The average radiographically measured Cobb angle of lumbar curvature was − 22.3 ± 1.4°; the average lumbar inclination was − 5.9 ± 0.6°.

### Apical rotation

**Fig. 2:**
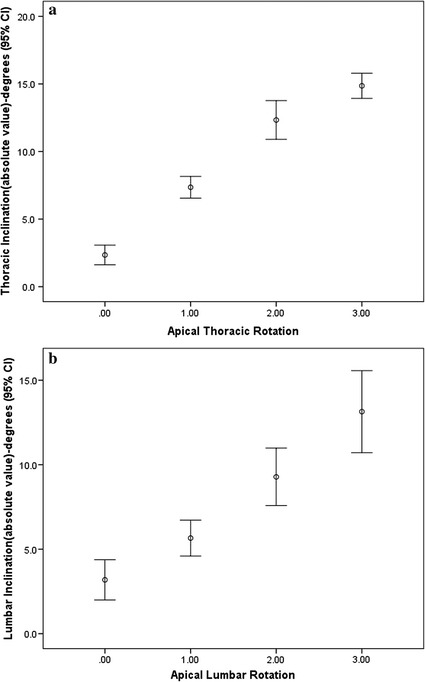
**a** The box plot graph of apical thoracic rotation and thoracic inclination. For the patients with Grade 0, Nash–Moe rotation was 2.3 (CI 1.6–3.1), for Grade 1 it was 7.4 (CI 6.6–8.2), Grade 2 it was 12.3 (CI 10.9–13.8) and Grade 3 it was 14.9 (CI 13.9–15.8). **b** The box plot graph of apical lumbar rotation and lumbar inclination. The average lumbar inclination for Grade 0 Nash–Moe rotation was 3.2 (CI 2.0–4.4), for Grade 1 it was 5.7 (CI 4.6–6.7), Grade 2 it was 9.3 (CI 7.6–11.0) and Grade 3 it was 13.1 (CI 10.7–15.6)

For the thoracic curve, there were 39 patients (38.6%) in Grade 0 Nash–Moe rotation, 42 patients (41.6%) in Grade 1, 6 patients (5.9%) in Grade 2 and 14 patients (13.9%) in Grade 3. The average absolute value of thoracic inclination for Grade 0 Nash–Moe rotation was 2.3 (CI 1.6–3.1); for Grade 1 it was 7.4 (CI 6.6–8.2), for Grade 2 it was 12.3 (CI 10.9–13.8) and for Grade 3 it was 14.9 (CI 13.9–15.8) (Fig. 2). The increase in inclination corresponded with the increase in rotational grading.

For the lumbar curve, there were 27 patients (26.7%) in Grade 0 Nash–Moe rotation, 35 patients (34.7%) in Grade 1, 25 patients (24.8%) in Grade 2 and 14 patients (13.9%) in Grade 3. The average absolute value of lumbar inclination for Grade 0 Nash–Moe rotation was 3.2 (CI 2.0–4.4), for Grade 1 it was 5.7 (CI 4.6–6.7), Grade 2 it was 9.3 (CI 7.6–11.0) and for Grade 3 it was 13.1 (CI 10.7–15.6). The increase in inclination also corresponded with the increase in rotational grading.

### Mathematical formulas

#### One-parameter formula

**Fig. 3:**
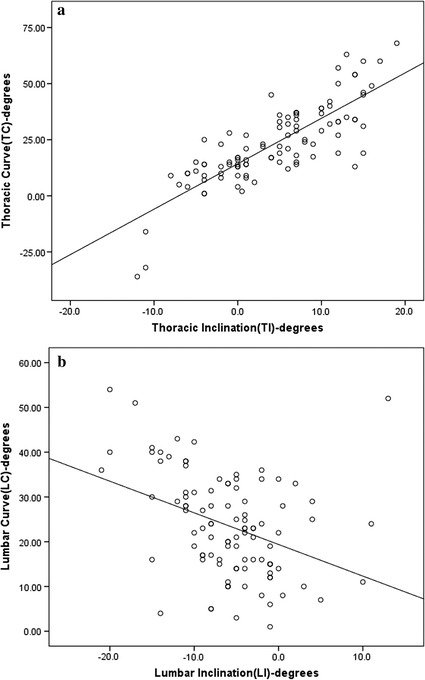
**a** The distribution of thoracic curve against thoracic inclination is inferred by simple linear regression. The r value is 0.813, which is statistically significant (*p* = 0.001). **b** The distribution of lumbar curve against lumbar inclination is inferred by simple linear regression. The r value is 0.409, which is statistically significant (*p* = 0.001)

Through the least squares method, the best a and b variables of the linear regression equation y = ax + b were determined. Thus, there were two different mathematical formulas to predict the thoracic and lumbar Cobb angles, respectively. For the thoracic curve, the formula was TC = 2.0 TI + 14.3 (TC = thoracic predicted Cobb angle; TI = thoracic scoliometer value, which represented thoracic inclination). For the lumbar curve, the formula was LC = 0.9 LI − 16.9 (LC = lumbar predicted Cobb angle; LI = lumbar scoliometer value, which represented lumbar inclination). The average predicted thoracic and lumbar Cobb angles were 23.3 ± 1.4 and − 22.3 ± 1.7, respectively. The thoracic and lumbar scoliometer values were statistically significantly correlated with the respective thoracic and lumbar Cobb angles (r = 0.813, p = 0.001 and r = 0.409, p = 0.001, respectively). There was a highly positive correlation between the predicted thoracic curve and the radiographically measured thoracic curve, while there was moderately positive correlation between the predicted lumbar curve values and the radiographically measured lumbar curve. The coefficient of the thoracic formula (a = 2.0) was larger than the coefficient of the lumbar formula (a = 0.9). The original value was plotted, and the estimated linear regression is also drawn in Fig. 3.

#### Two-parameter formula

**Fig. 4:**
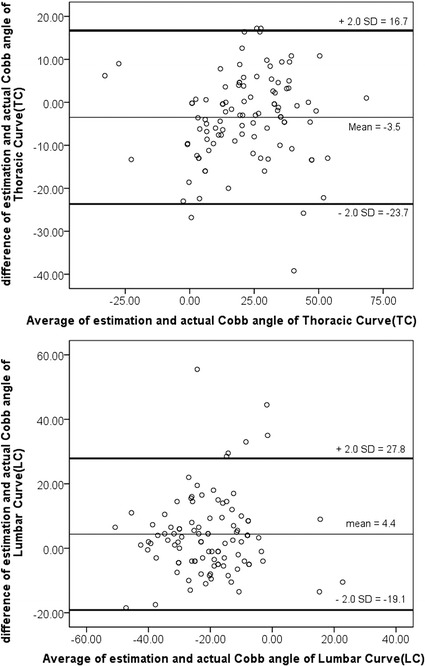
The Bland–Atman scatter plot quantifies the difference between the Cobb angles estimated by the two-parameter formulas and the Cobb angles measured from the radiographs versus the average of the two methods. The plot shows agreement between the two methods

For the two parameters, the multiple linear equation was y = ax_1_ + bx_2_ + c which c was set at 0. The adjusted formulas were TC = 2.6 TI − 1.4 LI for the thoracic curve and LC = − 1.5 TI + 2.0 LI for the lumbar curve. The average predicted thoracic and lumbar Cobb angles were 19.8 and − 18.4, respectively. The predicted thoracic and lumbar angles calculated by the two-parameter formulas were statistically correlated with the radiographically measured thoracic and lumbar angles (r = 0.931, p = 0.001 and r = 0.874, p = 0.001, respectively). The Bland–Atman scatter plot demonstrating the difference between the Cobb angles estimated by the two-parameter formulas and the Cobb angles measured from the radiographs versus the average of the two methods was shown in Fig. 4. Bland–Atman analysis showed agreement between the two methods. High positive correlation was proved when the two parameters were applied to predict thoracic and lumbar curves. Thus, the predicted Cobb angles calculated by the two-parameter formulas were more accurate than those calculated by the one-parameter formulas.

### Patient sample

**Fig. 5:**
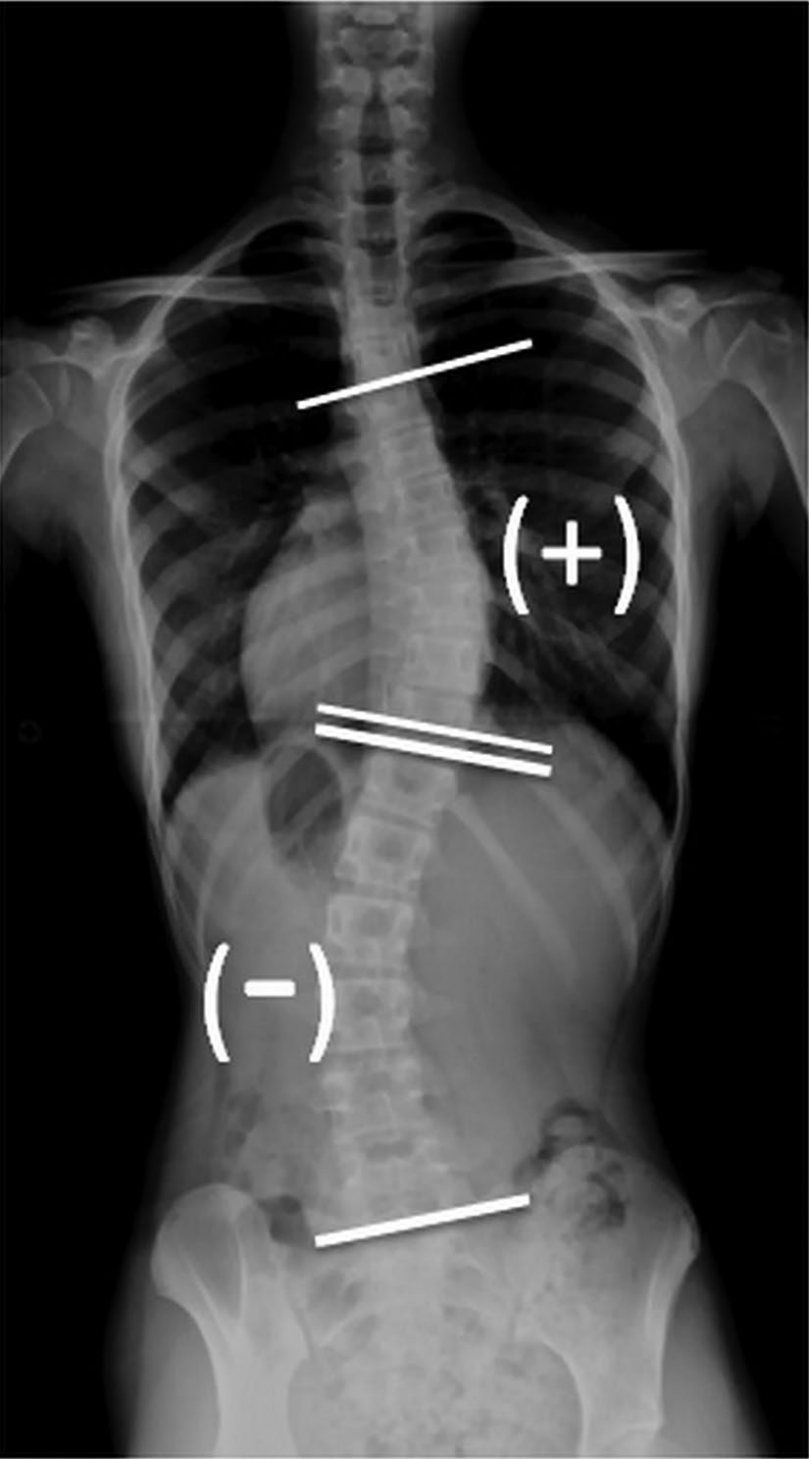
The whole spine AP view of a 14-year-old female patient. The measured thoracic curve was 37° (T5–T11), while the measured lumbar curve was − 30° (T12–L5). The predicted thoracic curve was 33.6°; the predicted lumbar curve was − 32.5° according to the two-parameter formulas

Figure 5 shows a 14-year-old AIS female. The thoracic inclination and lumbar inclination were measured by a scoliometer. The apical thoracic scoliometer value was 7, which was the right hump, and the apical lumbar scoliometer value was − 11, which was the left hump. For the one-parameter formula, the predicted thoracic Cobb angle was TC = 2.0 × 7 + 14.3 = 28.3, while the predicted lumbar Cobb angle was LC = 0.9 × (− 11) − 16.9 = − 26.8. For two-parameter formula, the predicted thoracic Cobb angle was TC = 2.6 × 7 − 1.4 × (− 11) = 33.6, while the predicted lumbar Cobb angle was LC = − 1.5 × 7 + 2.0 × (− 11) = − 32.5. The Cobb angles measured by whole spine AP view were 37° for the thoracic curve (T5–T11) and − 30° for the lumbar curve (T12–L5). The predicted thoracic and lumbar Cobb angles by the two-parameter formulas were closer to the measured thoracic and lumbar Cobb angles than those predicted by the one-parameter formulas.

## Discussion

### Vertebral rotation and trunk inclination

Vertebral rotation is a major challenge of idiopathic scoliosis because it is accompanied by lateral curvature of the spine. It is accepted that the severity of rib cage deformity is a function of the degree of vertebral rotation. Anatomically, vertebral rotation can cause deformity of the rib hump and rib depression, which can be detected on the surface of the back unless there is a vertebral deformity [17]. In this study, we excluded congenital, neuromuscular, traumatic and syndromic scoliosis.

The positive correlation between the rib hump and Cobb angle has been established in several studies [10, 11, 18]. Although Thulbourne and Gillespie [17] reported that there is no clear linear relationship between the rib hump and vertebral rotation, Cobb angle, and vertebral-rib angle, other studies [8, 11, 19] stated not only that vertebral rotation and rib hump have a close relationship but also that vertebral rotation is one of the factors causing scoliosis. The rib hump is also mentioned as the prognostic factor of scoliosis in a study by Duval-Beaupere [20]. It is more accurate to quantify the relationship between clinically measured inclination and vertebral rotation measured on axial computed tomography, but the method is not realistic due to issues of cost-effectiveness and fear of radiation exposure. Carlson et al. utilized the preoperative apical vertebra in the CT scans of sixteen females to detect the relationship between vertebral rotation and surface inclination and found that the clinically obtained angle of trunk inclination by scoliometer correlated well with coronal Cobb and CT-measured apical vertebral rotation in thoracic and thoracolumbar AIS curves preoperatively [21].

The current study has provided a more comprehensive understanding of adolescent idiopathic scoliosis as a three-dimensional spinal deformity, encompassing both lateral and rotational components. Instead of quantifying curve severity using the Cobb angle, vertebral rotation has become increasingly prominent in recent studies of scoliosis [22]. In the box-plot chart (Fig. 2), our study reveals that the greater the change in rotation of the vertebral body, the larger the degrees of inclination presented on the scoliometer, even if there is little overlap at each interval. As a result, the Nash–Moe rotation did reveal a positive proportional result in terms of scoliometer value.

### Scoliometer

Although the scoliometer was invented several decades ago, recent studies have shown that it still provides adequate intra-observer reliability values and very good inter-observer reliability [23]. In addition, Prowse et al. conducted a systematic review of the reliability and validity of inexpensive and easy clinical evaluation methods of adolescent idiopathic scoliosis. The scoliometer method with a one-parameter mathematical formula [10, 11] showed moderate to strong levels of evidence [12].

Although Adam’s forward bending test was previously useful for screening, the scoliometer can more precisely detect abnormality versus what appears to be normal during the forward bending test [24]. Literature reviews of vertebral rotation have confirmed the strong relationship between rib humps caused by vertebral rotation measured with a scoliometer and Cobb angle measured by standard posterior–anterior radiography [15]. It has been proven that the scoliometer is useful for indirectly calculating the Cobb angle through a specific formula. There have been two previous studies of mathematical formulas of Cobb angle predictions by noninvasive parameters, such as scoliometer value and height [10, 11]. Korovessis et al. [10] published a study describing how to predict scoliotic Cobb angle with one parameter using a scoliometer. The formulas were TC = 1.62 TI + 6.30 (TC = predicted thoracic angle, TI = apical thoracic scoliometer value) and LC = 1.58 LI + 7.36 (LC = predicated lumbar Cobb angle, LI = apical lumbar scoliometer value). The multi-regression relative values were 0.414 and 0.649, respectively. Sapkas et al. [11] reported a significantly strong correlation between scoliometer values and radiographic Cobb angles (r = 0.685). However, statistical analysis showed that radiographically measured Cobb angle and scoliometer values were correlated with one another (r = 0.215), but not significantly. Coelho et al. also reported a one-parameter formula and considered the correlation between scoliometer measurements and radiograph analysis to be good [23]. Table 1 shows the formulas and correlation values of the four studies compared with those of the current study.

**Table 1:** The mathematical formulas to calculate Cobb angles

Study	Parameter	Formula	Correlation value (r)
Korovessis et al. [10]	TI	TC = 1.62 TI + 6.30	0.414
	LI	LC = 1.58 LI + 7.36	0.649
Sapkas et al. [11]	TI	TC = 20.461 + 0.13 TI^2^	0.685
	LI, H	LC = 70.46 − 0.639 H + 5.707 LI	0.215
Coelho et al. [23]	ATR	C = − 6.3 + 2.7 ATR	0.7
The current study	TI, LI	TC = 2.6 TI − 1.4 LI	0.931
	TI, LI	LC = − 1.5 TI + 2.0 LI	0.874

TC = predicted thoracic Cobb angle, LC = predicted lumbar Cobb angle, TI = apical thoracic scoliometer value, LI = apical lumbar scoliometer value, H = body height, C = Cobb angle, ATR = axial trunk rotation

In our study, we considered that the severities of the thoracic curve and lumbar curve can influence one another. The compensation mechanism for spinal coronal balancing can explain this phenomenon. Based on our formulas, a greater degree of lumbar inclination will lead to a higher predicted degree of thoracic curvature. To balance the spine, a larger positive thoracic curve (right curve deemed positive) is needed to compensate for a larger negative lumber curve (left curve deemed negative). Similarly, a greater degree of thoracic inclination will lead to a higher predicted degree of lumbar curvature. A larger negative lumbar curve is needed to compensate for a larger positive thoracic curve. The inclination of the thoracic and lumbar humps measured by the scoliometer will influence the predicted thoracic and lumbar Cobb angles simultaneously. As a result, it is more accurate to use the two-parameter formulas to predict thoracic and lumbar Cobb angles.

### Limitations

There are several limitations in this study. First, the body mass indexes of the enrolled patients were not included in the formula. Body mass index may influence the accuracy of the formula [25]. However, the formula would become much more complicated and unrealistic to apply if too many parameters were taken into account. Second, all of the curves of the enrolled patients were right convex curves at the thoracic level and left convex curves at the thoracolumbar or lumbar levels. Third, patients who were diagnosed as having neuromuscular, congenital, traumatic or syndromic scoliosis were excluded. The manifestations of non-idiopathic scoliosis include less vertebral rotation and thus less rib hump. The Cobb angles calculated by scoliometer values were smaller than the measured Cobb angles. Finally, scoliometer measurement might have a slight interobserver and intraobserver variation. However, previous studies evaluating the interobserver and intraobserver reliability indicated adequate measurement reproducibility [15, 26]. All the scoliometer values in this study were measured by one single senior surgeon. Scoliometer can be a reliable noninvasive method for assessing spinal axial rotation when used by a single trained observer [26].

### Clinical application

In clinical practice, the two-parameter formulas can be applied for scoliosis screening in schools. Using these two-parameter formulas, the scoliometer can play a vital role in the prediction of scoliotic curvature. Using a scoliometer and these two-parameter formulas can likely replace X-rays in screening for idiopathic scoliosis with calculated Cobb angles less than 20° in the first clinical visit, and with calculated Cobb angles less than 40° in the follow-up visit if brace treatment is not necessary. This can not only save medical costs but also allay concerns over radiation exposure. In addition, the scoliometer is more easily distributed to healthcare providers who are not equipped with X-rays, especially in rural areas or under-developed countries. The predicted Cobb angles calculated by the formulas with two parameters are closer to the radiographically measured Cobb angles than those with one parameter.

## Conclusions

In this study, we demonstrated that the predicted Cobb angles calculated by the formulas with two parameters are closer to the radiographically measured Cobb angles than those with one parameter. Combined with the thoracic and lumbar scoliometer values, it is more accurate in predicting the thoracic and lumbar Cobb angles, respectively. We considered that the magnitude of the thoracic curve and lumbar curve can simultaneously influence the readouts of the scoliometer at thoracic and lumbar humps. The two-parameter formulas are recommended for screening or follow-up of patients with idiopathic scoliosis because the results are more accurate and the method is cost-effective. Additionally, there is less risk of patients being exposed to radiation.
